# 3D scene reconstruction from body‐worn camera video using 3DF Zephyr

**DOI:** 10.1111/1556-4029.70283

**Published:** 2026-02-05

**Authors:** Yuening Chen, Eugene Liscio

**Affiliations:** ^1^ Department of Forensic Science University of Toronto Mississauga Mississauga Ontario Canada; ^2^ ai2‐3D Forensics Woodbridge Ontario Canada

**Keywords:** 3D reconstruction, 3DF Zephyr, body‐worn video, digital forensics, forensic measurement validation, photogrammetry, point clouds

## Abstract

Body‐worn cameras document crime scenes during initial law enforcement response, yet their potential for forensic reconstruction has not been empirically validated. Despite expanding global adoption, recorded video primarily serves qualitative documentation rather than quantitative measurement applications. This study empirically evaluated three‐dimensional (3D) reconstruction accuracy from body‐worn camera video to assess its feasibility for feature measurement. Three Axon camera models—Body 2 (AB2), Flex 2 (AF2), and Body 3 (AB3)—were tested in an outdoor parking lot, with each model recording five videos at both 720P and 1080P resolutions (*n* = 30). Videos were recorded under controlled experimental conditions to achieve optimized documentation scenarios. Videos were processed using 3DF Zephyr photogrammetry software to create 3D reconstructions, then compared against Faro Focus S350 laser scanner ground truth at three distances: long (12.48 m), medium (2.42 m), and short (0.24 m). One‐sample t‐tests revealed significant differences between AF2 measurements and ground truth (*p* < 0.05), with a maximum mean error of 14.42 cm at 720P for long distances. AB2 and AB3 showed no significant differences from the ground truth at both resolutions across all validation distances (*p* ≥ 0.05). Two‐sample *t*‐tests demonstrated no significant differences between resolutions (*p* ≥ 0.05). Single‐factor ANOVAs indicated significant differences between camera models (*p* < 0.05). Resolution did not affect measurement accuracy under the conditions of the controlled methodology and internal software interpolation. These best‐case results demonstrate that with deliberate documentation protocols, accurate 3D reconstruction from body‐worn camera video is achievable for forensic applications.

**Highlights**

- Initial empirical validation shows body‐worn cameras enable accurate forensic scene reconstruction.
- Video‐based 3D reconstruction provides quantitative measurements beyond visual documentation.
- Resolution did not affect measurement accuracy under controlled methods with software interpolation.
- Camera models showed significant differences: AF2 maximum mean error 14.42 cm vs. AB2/AB3 <5.50 cm.
- Best‐case testing establishes a baseline for future body‐worn camera 3D reconstruction practice.

## 1 INTRODUCTION

The use of body‐worn cameras by law enforcement personnel has experienced rapid global expansion since the early 2000s, with Australia and the United Kingdom leading the way as early adopters [1]. These small and portable devices are typically positioned at the neck or chest level to record real‐time documentation of officer interactions with the public and at crime scenes [2]. By 2019, over 70% of police forces in the United Kingdom had integrated body‐worn cameras and were moving toward complete implementation [1]. The adoption of body‐worn cameras by law enforcement personnel in the United States also experienced a significant increase between 2013 and 2016. During this period, the proportion of local police departments utilizing these devices nearly doubled, with 60% having fully deployed them by 2016, along with 49% of sheriff's offices [1]. Moreover, by 2016, 86% of general‐purpose law enforcement agencies in the United States that had acquired body‐worn cameras either had formal policies governing their use in place or were in the process of developing such policies, highlighting the institutionalization of this technology into standard operating procedures [1]. The Canadian government also supports the implementation of body‐worn cameras, positing that these devices can increase trust between police and communities. This rationale is founded on the premise that the cameras record unbiased and objective video evidence of interactions between police and communities, thus providing a transparent and verifiable record that enhances accountability of law enforcement practices [2, 3]. The Canadian government has initiated a plan to issue between 10,000 and 15,000 body‐worn cameras to contract and federal police officers who interact with communities, with the ultimate aim of equipping all frontline officers with these devices [3].

The implementation of body‐worn cameras represents a significant transformation in police operational tactics, particularly in the preliminary documentation and inspection of crime scenes [4]. This implementation underscores the increasing significance of the digital domain in forensic science, as recorded video can serve as crucial direct evidence in legal and disciplinary proceedings to support or challenge the actions of police officers [5, 6]. This evidential role necessitates a comprehensive examination of the authenticity and constraints of video as legal evidence, as well as exploration of potential values and applications beyond basic video documentation [4]. One such application is three‐dimensional (3D) reconstruction from video data. However, research systematically validating dimensional measurement accuracy from body‐worn camera video reconstructions remains absent from the literature. A systematic literature review documents 258 papers on 3D forensic reconstruction applications, yet these studies predominantly use photogrammetry of static photographs or laser scanning for scene documentation, with video‐based reconstruction representing a minimal subset limited to surveillance cameras or drones rather than body‐worn cameras [7, 8]. One study specifically addressing body‐worn camera video developed a methodology for creating what they term “3D images” of crime scenes, but provided only a single accuracy test yielding 5% error while explicitly stating this result “needs to be examined in every case individually” and “is not valid as it can differ in other cases,” thus acknowledging the absence of systematic validation [9]. Commercial software has attempted measurement extraction from body‐worn camera video only through single‐frame analysis to produce 2D scene diagrams, eliminating depth information essential for 3D feature measurement while lacking peer‐reviewed validation [10].

Therefore, this study aims to explore the feasibility of utilizing video obtained from body‐worn cameras to create accurate and reliable 3D reconstructions using 3DF Zephyr photogrammetry software for forensic applications. The main purpose is to validate the accuracy of 3D reconstructions from body‐worn camera video against high‐resolution laser scanner ground truth to establish a scientific basis for their application in legal contexts. This validation uses a best‐case scenario approach with deliberate optimization of recording conditions to assess the accuracy achievable under these controlled conditions rather than replicate typical field documentation practices. The significance of this controlled validation lies in establishing whether body‐worn camera video can enable three fundamental forensic reconstruction applications:

1. Immediate crime scene capture and preservation: In actual police operations, body‐worn cameras record initial contact with undisturbed crime scenes, capturing video documentation as officers first arrive and move through the scene. This initial contact video differs from formal photographic documentation, as the camera records based on incidental officer movement rather than deliberate evidence‐focused capture [9]. To determine whether body‐worn cameras can support dimensional reconstruction, this study tests deliberate recording techniques under controlled conditions to establish a performance baseline. If reconstruction fails under optimized circumstances, field applications using incidental recording would be infeasible. The study utilizes an outdoor parking lot as a controlled test environment with measurable architectural features, avoiding legal and privacy constraints associated with actual law enforcement video. This approach evaluates whether body‐worn cameras can preserve dimensional features and spatial relationships sufficient for quantitative forensic reconstruction beyond qualitative visual review.
2. Large‐scale feature measurement: Following the feasibility assessment, this study evaluates whether 3D point clouds reconstructed from body‐worn camera video enable accurate dimensional measurements and preserve spatial relationships between scene features. The validation extracts distances between fixed architectural reference points within the reconstructed point cloud and compares these against corresponding laser scanner ground truth data to quantify measurement errors. The study examines large‐scale architectural features rather than small evidence items, recognizing that body‐worn cameras capture scenes at distances determined by officer movement, while small evidence documentation follows separate close‐range photography procedures. This approach determines whether body‐worn cameras can capture scene‐level spatial information with reasonable measurement errors for forensic applications.
3. Temporal scene comparison: Body‐worn cameras record crime scenes during first officer response, preserving spatial information before investigative activities or environmental factors alter original conditions. Formal forensic documentation occurs later in time after forensic technicians arrive. This temporal gap between initial response and formal documentation represents a critical period where scene modifications could occur. Without validated measurement accuracy from body‐worn cameras, initial response recording would remain qualitative observation rather than quantifiable measurement. The measurement accuracy validated in this study thus determines whether body‐worn camera video can provide dimensional documentation when laser scanning is not performed or when original scene conditions cannot be preserved. Although temporal comparisons are not examined in this study, assessing achievable measurement accuracy establishes the foundation for future research comparing the initial response video with formal documentation.

## 2 MATERIALS AND METHODS

**FIGURE 1:**
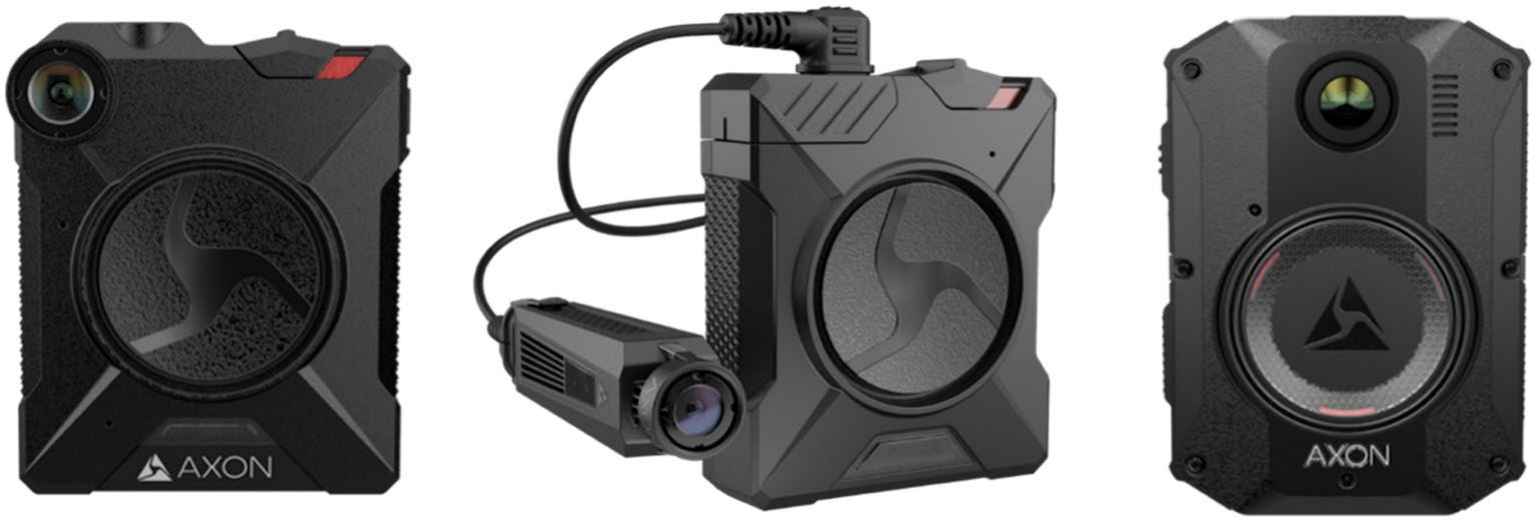
Axon body‐worn camera models used in this study. From left to right: Axon Body 2 (AB2), Axon Flex 2 (AF2), and Axon Body 3 (AB3). The AB2 and AB3 are chest‐mounted units, while the AF2 uses a separate camera head and controller unit design. These models represent distinct generations of body‐worn camera technology with specifications (field of view: AF2 120°, AB2 143°, AB3 146°) that could influence reconstruction outcomes [11].

This study used controlled video recording and photogrammetric processing procedures for dimensional reconstruction and measurement accuracy validation from body‐worn camera video. Three Axon camera models were tested from an available pool of 15 units documented in Table 1 (5 units per model): Axon Body 2 (AB2), Axon Flex 2 (AF2), and Axon Body 3 (AB3) (Figure 1) [11]. These models represent different technological generations with distinct hardware designs and firmware implementations. AB2 operates on firmware version 1.27.7 across all units as a chest‐mounted integrated design, AF2 runs firmware versions 0.5.9 and 0.7.3 with a modular camera head and controller configuration allowing flexible mounting positions, and AB3 uses firmware version 1.19.45 as a chest‐mounted integrated design with improved low‐light performance and image stabilization representing the most advanced model available for this study (Table 1) [11]. All three models support identical recording resolutions (1080P, 720P, 480P), with 720P configured as the default setting for most law enforcement agencies to balance video quality and storage requirements [11]. The models differ in their fields of view: AF2 (120° diagonal, 102° horizontal, 55° vertical), AB2 (143° diagonal), and AB3 (146° diagonal) [11]. All three models use CMOS sensors with fixed‐focus wide‐angle lenses to achieve these fields of view.

**TABLE 1:** Technical specifications of body‐worn cameras available for the study. Documentation includes model designation, serial numbers, unit identifiers, and firmware versions for 15 available units (5 units per model). Firmware versions varied within AF2 units (0.5.9 and 0.7.3) while remaining consistent within AB2 (1.27.7) and AB3 (1.19.45) models. This firmware variation is documented as a technical difference between units and could contribute to performance differences observed in the measurement results. The 30 test videos were recorded using selected units from this pool.

Model	Serial no.	Camera name	Firmware
AF2	X84202072	Flex2‐5	0.7.3
AF2	X84148529	Flex2‐4	0.7.3
AF2	X84152678	Flex2‐3	0.5.9
AF2	X84149623	Flex2‐2	0.7.3
AF2	X84146891	Flex2‐1	0.7.3
AB3	X60C06425	Body3‐5	1.19.45
AB3	X60C00286	Body3‐4	1.19.45
AB3	X60C07815	Body3‐3	1.19.45
AB3	X60C06555	Body3‐2	1.19.45
AB3	X60C08175	Body3‐1	1.19.45
AB2	X81662398	Body2‐5	1.27.7
AB2	X81664662	Body2‐4	1.27.7
AB2	X81666383	Body2‐3	1.27.7
AB2	X81666234	Body2‐2	1.27.7
AB2	X81666016	Body2‐1	1.27.7

**FIGURE 2:**
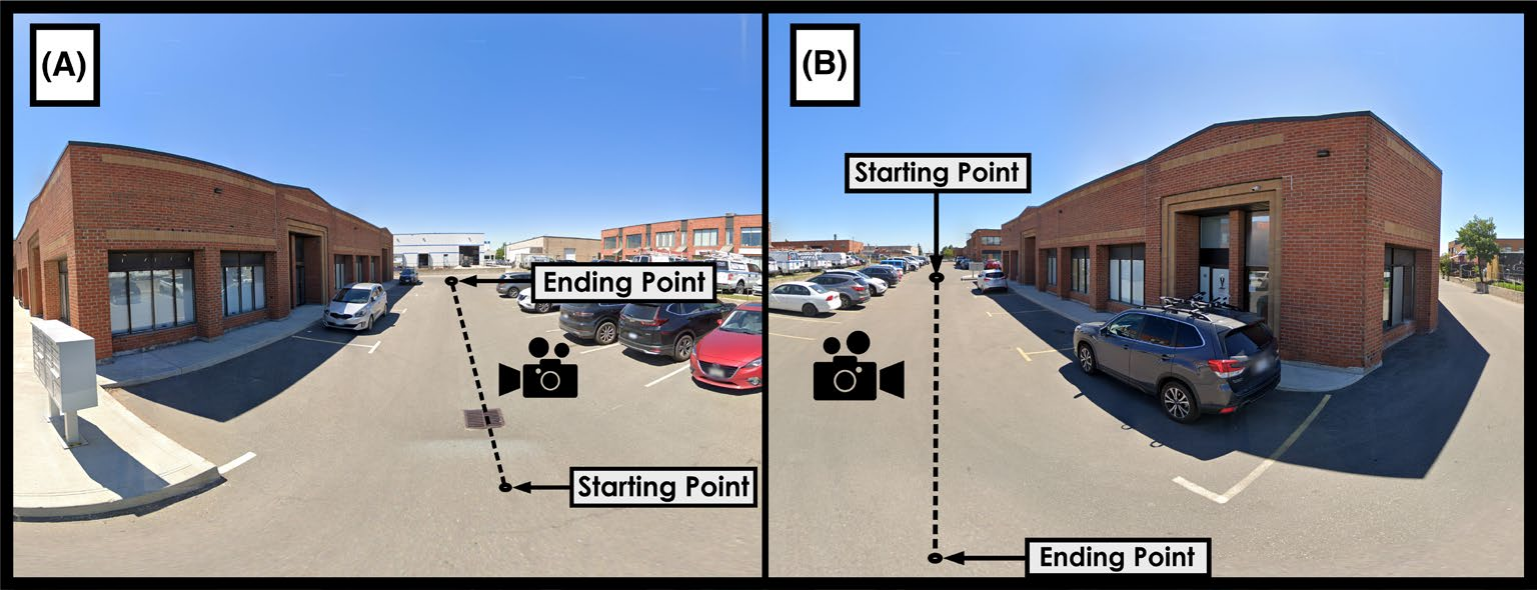
Documentation of the outdoor parking lot test area and 30 m data collection path. (A) View from the starting point showing the building facade. (B) View from the endpoint showing the complete test area from the opposite direction. The dashed lines (added to both photographs) indicate the path along which the researcher walked sideways while maintaining the camera oriented toward the building facade. Although lens distortion affects the photograph representations, the actual walking path was parallel to the building facade. This controlled sideways walking method ensured consistent camera‐to‐subject distance throughout video capture for optimal photogrammetric reconstruction [12].

From the available camera pool, each of the three camera models recorded five videos of 35–45 s duration at an outdoor parking lot measuring 30 m in length (Figure 2). This duration corresponded to the time required for the researcher to walk at a constant moderate speed in a straight line across the testing area, minimizing motion blur while ensuring comprehensive coverage of the test location. The researcher walked sideways along the predetermined path with cameras handheld at chest height (1.2 m from ground level, researcher height 1.6 m), maintaining camera orientation toward the building facade throughout recording (Figure 2). This sideways walking technique enabled visual contact with the building while preserving a consistent camera‐to‐subject distance. While this handheld recording method introduces minor variation in camera orientation and position due to manual control rather than mechanical stabilization from tripods or mounting equipment, it represents practical field documentation achievable when officers exercise deliberate camera control with conscious attention to stability and comprehensive feature capture during initial scene response.

To examine resolution effects on reconstruction feasibility and feature measurement accuracy, this study tested two recording resolutions available on all camera models: high resolution at 1920 × 1080 pixels (1080P) and medium resolution at 1280 × 720 pixels (720P). These resolutions were selected because they represent standard law enforcement configurations: 720P is the manufacturer‐recommended default that balances recording quality and storage requirements, while 1080P provides maximum available resolution for body‐worn cameras [11]. The 480P setting was excluded from testing as it is designated for extended battery life and faster upload speeds at the expense of recording quality, conflicting with the best‐case scenario approach used in this controlled validation [11]. Testing both resolutions across all camera models produced 30 videos in total (5 videos × 2 resolutions × 3 camera models). These videos were recorded under controlled conditions to minimize variables affecting reconstruction outcomes. Recording conditions beyond camera movement included the following controlled parameters: (1) Lighting conditions: All recordings occurred during clear weather between 10 a.m. to 3 p.m. to ensure consistent natural illumination. Recording sessions were postponed during rain, heavy cloud cover that created uneven lighting, or periods of direct sunlight that caused overexposure of the building facade. This temporal and weather control maintained uniform lighting conditions across all 30 videos. (2) Environmental interference: Videos containing visual obstructions were discarded and re‐recorded. Obstructions included pedestrians or vehicles passing between the camera and the building. Each recording required an uninterrupted 35–45 s capture of the complete test area to ensure continuous scene documentation. (3) Recording quality standards: Multiple recording attempts were conducted for each video until specific quality criteria were met. Acceptable videos demonstrated minimal motion artifacts from camera shake, consistent exposure throughout the recording duration without auto‐adjustment fluctuations, and comprehensive capture of architectural features including windows, doors, and structural edges necessary for control point identification. Videos failing any criterion were re‐recorded until all standards were satisfied. These control measures ensure that observed limitations reflect technological constraints rather than avoidable environmental or procedural deficiencies.

**FIGURE 3:**
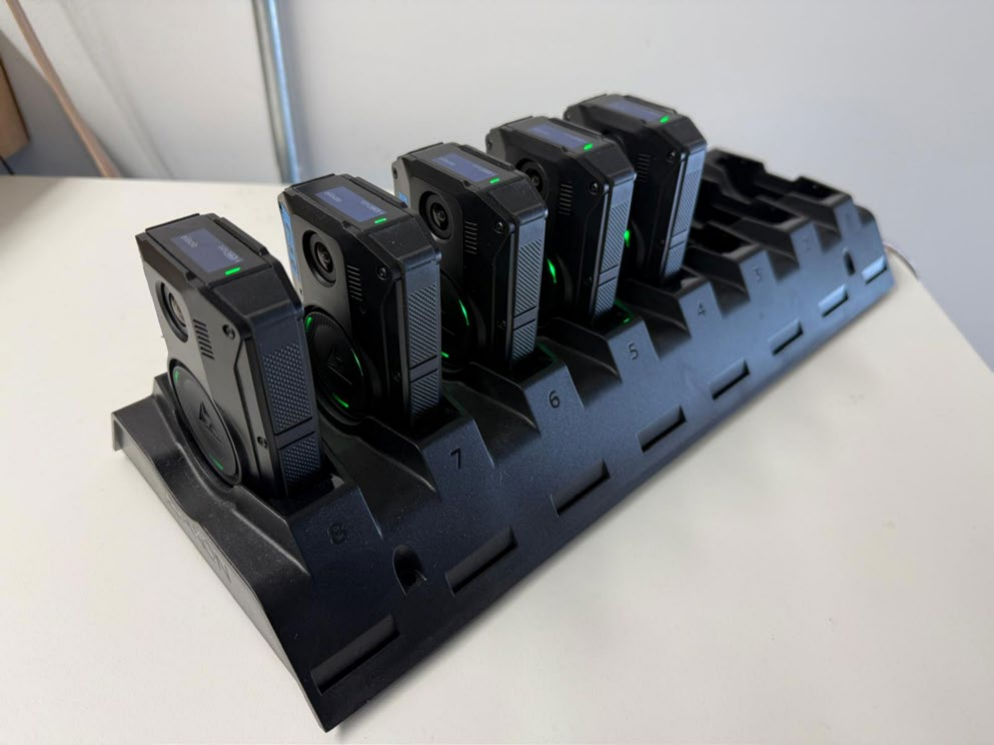
Axon body‐worn cameras in the docking station following data collection. The docking process transfers recorded videos to the Axon evidence management system while recharging batteries. Green LED indicators confirm successful video upload. Each of the three camera models (AB2, AF2, AB3) recorded five videos at two resolutions (720P and 1080P), resulting in 10 videos per model. All 30 videos were retrieved through this docking process and exported as MP4 files for processing in 3DF Zephyr photogrammetry software [13].

Following data collection, all 30 videos required transfer from the body‐worn cameras for photogrammetric processing. Each camera was placed into an Axon docking station which serves dual functions: establishing network connectivity to upload videos to the Axon evidence management system while charging the camera battery (Figure 3) [13]. Green LED indicators confirmed successful data transfer. Once uploaded to the Axon evidence management system, all 30 videos were exported as MP4 files for processing in 3DF Zephyr photogrammetry software [13]. For 3D reconstruction from these video files, photogrammetry was selected as the processing method because it provides a cost‐effective and non‐invasive technique to transform 2D video frames into 3D spatial coordinates. This technique operates by identifying common features across multiple viewing angles and calculating their 3D positions through triangulation [14].

**FIGURE 4:**
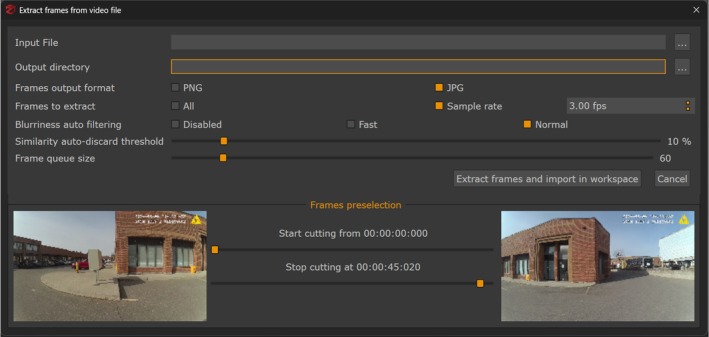
3DF Zephyr photogrammetry software interface showing frame extraction parameters. The software is set to extract frames at a rate of 3 frames per second with a 10% similarity threshold. Axon cameras encode video using a 30‐frame GOP structure (one I‐frame and 29 P‐frames). As the software does not provide an option to differentiate frame types during extraction, this results in a mixed collection of I‐frames (full image data) and P‐frames (difference data). Frames that are too blurred or exceed the 10% similarity threshold are automatically discarded. This frame extraction constitutes the first step in photogrammetric reconstruction, determining which frames proceed to sparse point cloud generation.

To begin the photogrammetric process, 3DF Zephyr extracted individual frames from each video at a rate of 3 frames per second, with automatic discard of frames exhibiting motion blur or exceeding 10% similarity threshold (Figure 4). This extraction process must be understood within the context of how body‐worn cameras encode video data. Video compression uses Group of Pictures (GOP) structures containing three frame types: I‐frames (intra‐coded frames with complete image data), P‐frames (predicted frames storing differences from previous frames), and B‐frames (bi‐directional frames referencing both previous and future frames) [15]. Axon body‐worn cameras encode video using a GOP of 30 frames consisting of one I‐frame followed by 29 P‐frames without B‐frames. At 30 frames per second recording, the 35‐s videos contained 1050 frames total (35 I‐frames and 1015 P‐frames), while 45‐second videos contained 1350 frames total (45 I‐frames and 1305 P‐frames). However, video compression analysis, which is the process of identifying and cataloging frame types within the video file, was not performed prior to frame selection [15]. Such analysis would require separate video analysis software to examine the encoded video structure and identify I‐frame positions before extraction. Instead, 3DF Zephyr extracted frames at the specified 3 frames per second setting using its automated extraction algorithm. The software processes each extracted frame as an independent image without distinguishing between frame types, and it reports only whether extraction succeeded or failed, without identifying which frame types were selected or how many frames were rejected for blur or similarity violations. Therefore, the extracted datasets contain a mixed distribution of I‐frames and P‐frames in unknown proportions.

**FIGURE 5:**
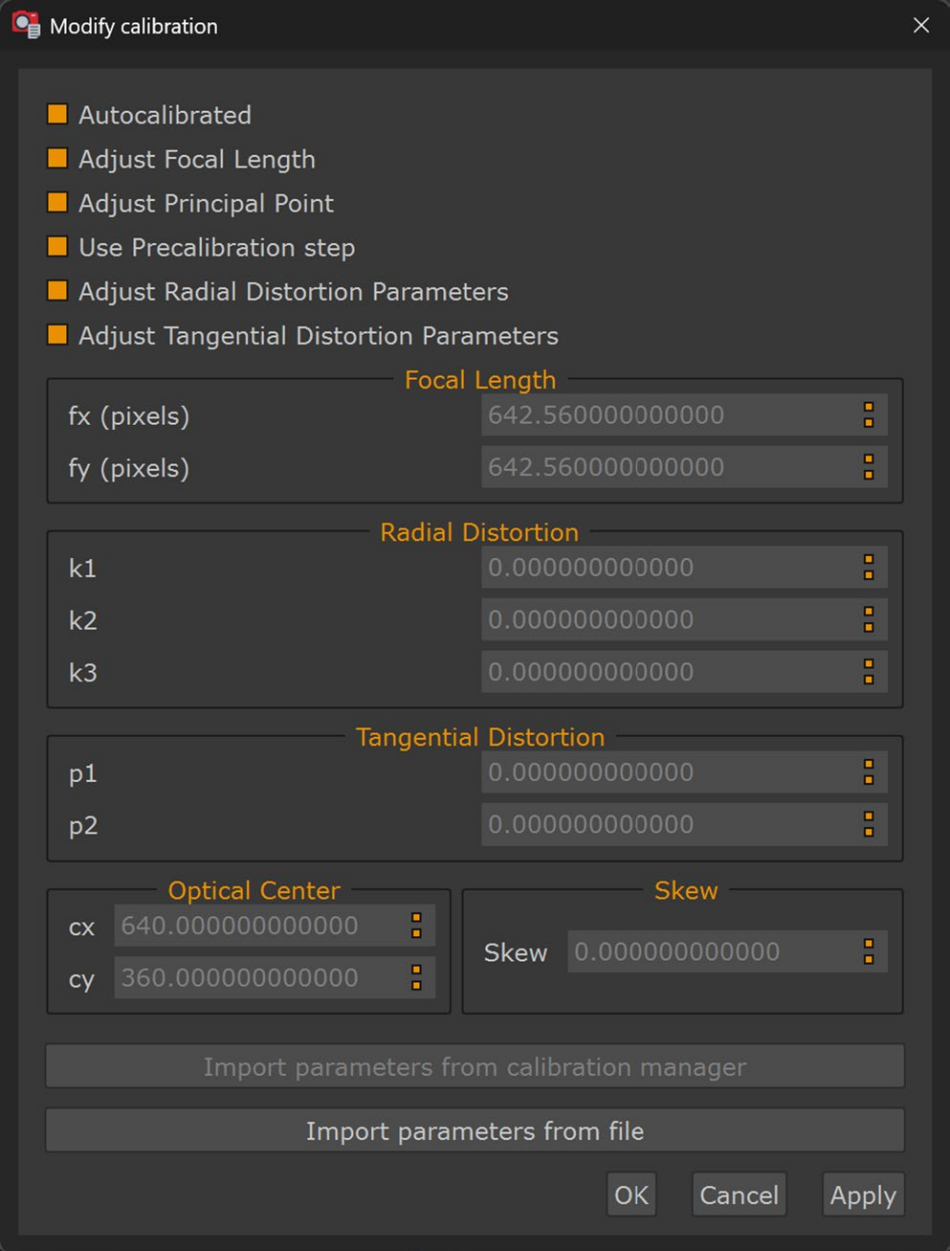
3DF Zephyr camera calibration interface with autocalibration selected. The software provides options for manual calibration input or automated calibration from extracted frames. All 30 videos were processed using automated calibration, which estimates intrinsic camera parameters from the video frames without requiring calibration charts or pre‐calibration procedures. This calibration step occurs after frame extraction and before sparse point cloud generation, establishing camera parameters for photogrammetric reconstruction.

After successful frame extraction, 3DF Zephyr presented camera calibration options, allowing selection between manual parameter input or automated calibration from the extracted frames (Figure 5). Camera calibration determines the mathematical parameters that map 3D spatial coordinates to their corresponding 2D pixel positions in video frames [16]. For Axon body‐worn cameras, this calibration process corrects the significant barrel distortion caused by wide‐angle lenses (120°–146° field of view). Without correcting this distortion, pixel measurements from video frames cannot translate to accurate real‐world distances, preventing validation against laser scanner ground truth. All 30 videos were processed using automated calibration, where the software calculated focal length, principal point, and distortion coefficients from the extracted frames themselves. This approach eliminated the need for calibration charts or pre‐calibration procedures, making it applicable to forensic scenarios where original equipment parameters are unavailable.

**FIGURE 6:**
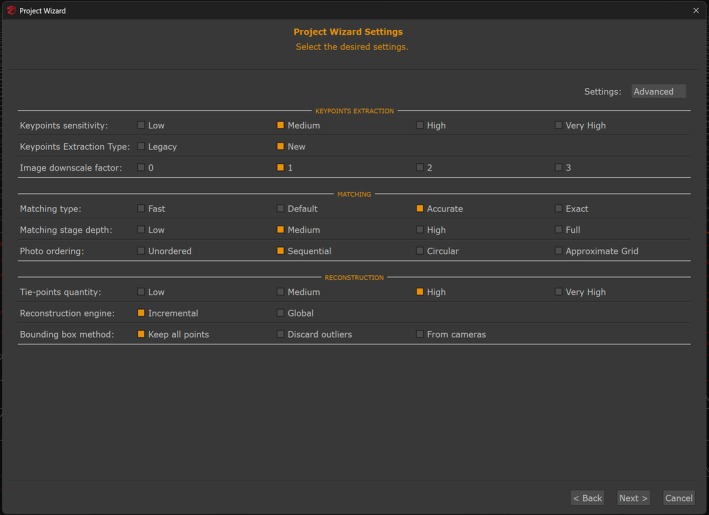
3DF Zephyr interface for sparse point cloud generation. The software identifies overlapping image pairs and extracts tie points to calculate 3D coordinates from the calibrated 2D frames. The software processes all valid frame pairs containing sufficient common tie points to create the sparse point cloud. This reconstruction phase establishes the initial 3D spatial relationships between the calibrated frames. Identical settings were applied to all 30 videos to ensure methodological consistency across camera models and resolutions. The process generated the initial 3D point cloud structure used for later dense reconstruction and mesh generation.

**FIGURE 7:**
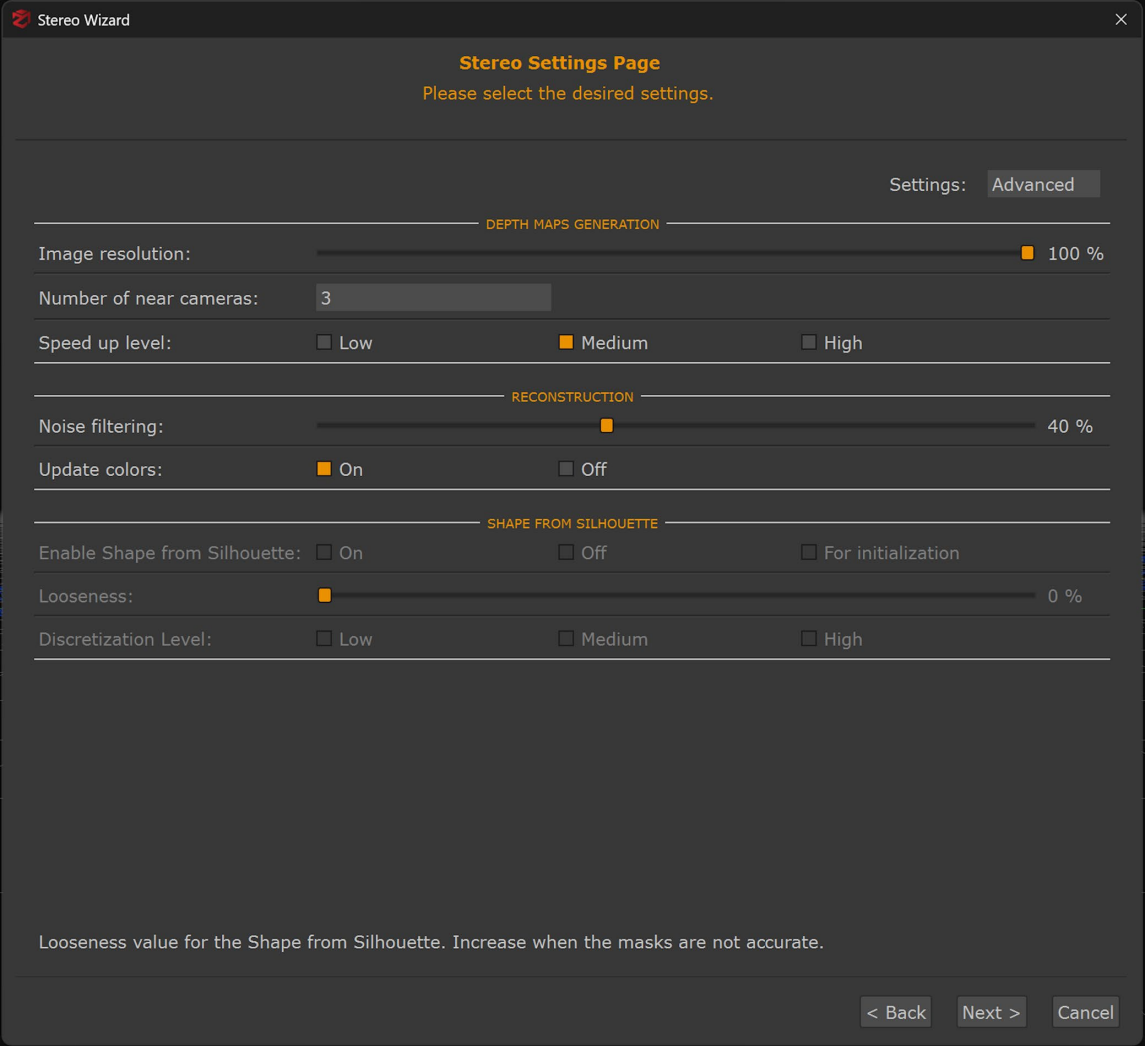
3DF Zephyr interface for dense point cloud generation. The software constructs depth maps for overlapping image pairs and merges them to create a dense point cloud. This process multiplies point density from the sparse point cloud, filling gaps between initial tie points with additional surface detail. The software applies 40% noise filtering to remove outlier points and updates color information from the original frames, enabling visual identification of architectural features for control point selection. Dense point cloud generation is critical for accurate feature measurement as it provides the surface detail necessary for identifying control points and measuring distances. Identical settings were applied to all 30 videos to maintain methodological consistency. The resulting dense point cloud provides sufficient surface resolution for mesh creation and measurement validation.

**FIGURE 8:**
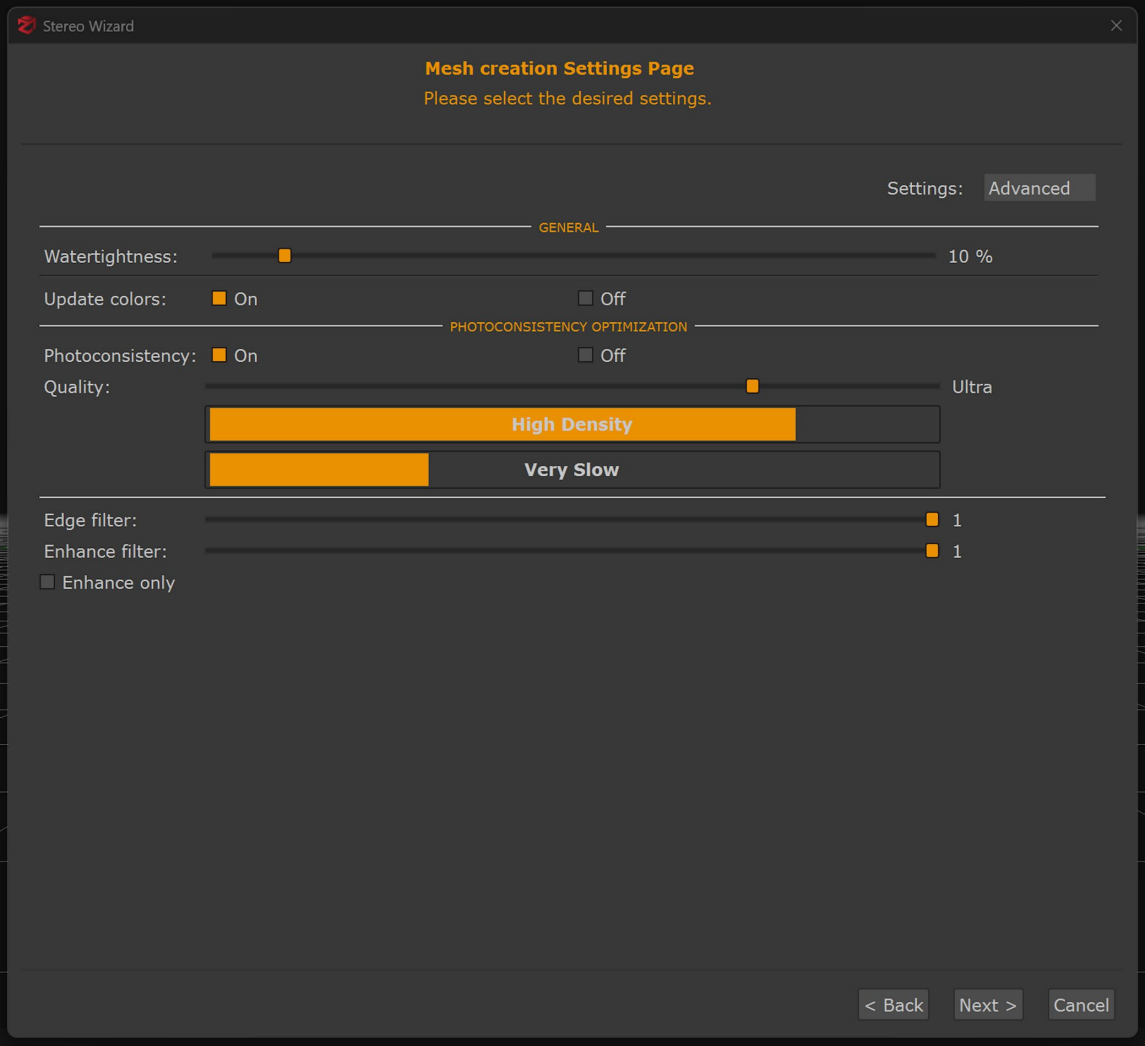
3DF Zephyr interface for mesh generation. The software converts the dense point cloud into a triangulated surface model. Settings include high‐density quality to maximize triangle count for architectural details, photoconsistency optimization to ensure texture consistency across overlapping images, and 10% watertightness to preserve actual openings (windows, doors) rather than forcing surface closure. Edge filtering reduces noise on flat surfaces while maintaining sharp boundaries at architectural features. The mesh generates a continuous textured surface from the dense point cloud data, completing the three‐step photogrammetric reconstruction process.

With calibration parameters established, the software processed the extracted frames through three sequential reconstruction phases: (1) Sparse point cloud generation identified and matched tie points across overlapping frame pairs to calculate initial 3D coordinates through triangulation (Figure 6). The software detected common features between frames with sufficient overlap to establish the initial 3D point cloud structure and relative camera positions for each frame. (2) Dense point cloud generation computed depth maps for each frame using information from overlapping frames, then merged these depth maps to increase point density from the sparse point cloud (Figure 7). The software applied 40% noise filtering to remove outlier points while preserving architectural features and color information necessary for control point identification. (3) Mesh generation converted the dense point cloud into a continuous triangulated surface model (Figure 8). The software applied surface reconstruction algorithms to connect neighboring points into triangular faces. Processing parameters included high‐density quality for architectural detail capture, photoconsistency optimization for texture coherence, and 10% watertightness to preserve actual openings (windows, doors) while filling gaps smaller than this threshold. All 30 videos received identical reconstruction parameters throughout these three phases to ensure methodological consistency across camera models and resolutions. This three‐phase reconstruction produced measurable 3D models from the extracted video frames, completing the photogrammetric processing before measurement validation.

Upon completion of reconstruction for all 30 recorded videos, dimensional validation required two critical components: scaling the dimensionless photogrammetric models to real‐world measurements and establishing an accurate reference standard for comparison. A Faro Focus S350 laser scanner was selected to capture the reference model of the building facade based on its established use in forensic documentation and verifiable measurement specifications [17]. The Faro Focus S350 achieves ±1 mm ranging error at both 10 m and 25 m distances with 19 arcsecond angular accuracy for vertical and horizontal measurements. These specifications combine to produce 3D position accuracy of 2 mm at 10 m and 3.5 mm at 25 m, with uncertainty increasing by only 0.1 mm per m beyond 25 m [17]. The scanner operates at speeds up to 976,000 points per second with a 300° vertical and 360° horizontal field of view, providing rapid and near‐complete scene coverage despite a 60° blind spot beneath the unit [17]. The reference data collection in this study required eight scanning positions to document the entire building facade. Registration of these scans confirmed operational performance within manufacturer specifications. This process achieved maximum point error of 3.0 mm and mean point error of 1.9 mm with 78.2% minimum overlap between adjacent scans. The mean error of 1.9 mm falls within the specified 2 mm accuracy at 10 m, confirming that actual scanner performance matched documented specifications. These performance metrics establish the reference model as suitable ground truth, with millimeter‐level mean registration error providing the dimensional accuracy necessary for validation purposes. With the reference model validated at this precision level, it could serve as the dimensional baseline for scaling the photogrammetric reconstructions.

**FIGURE 9:**
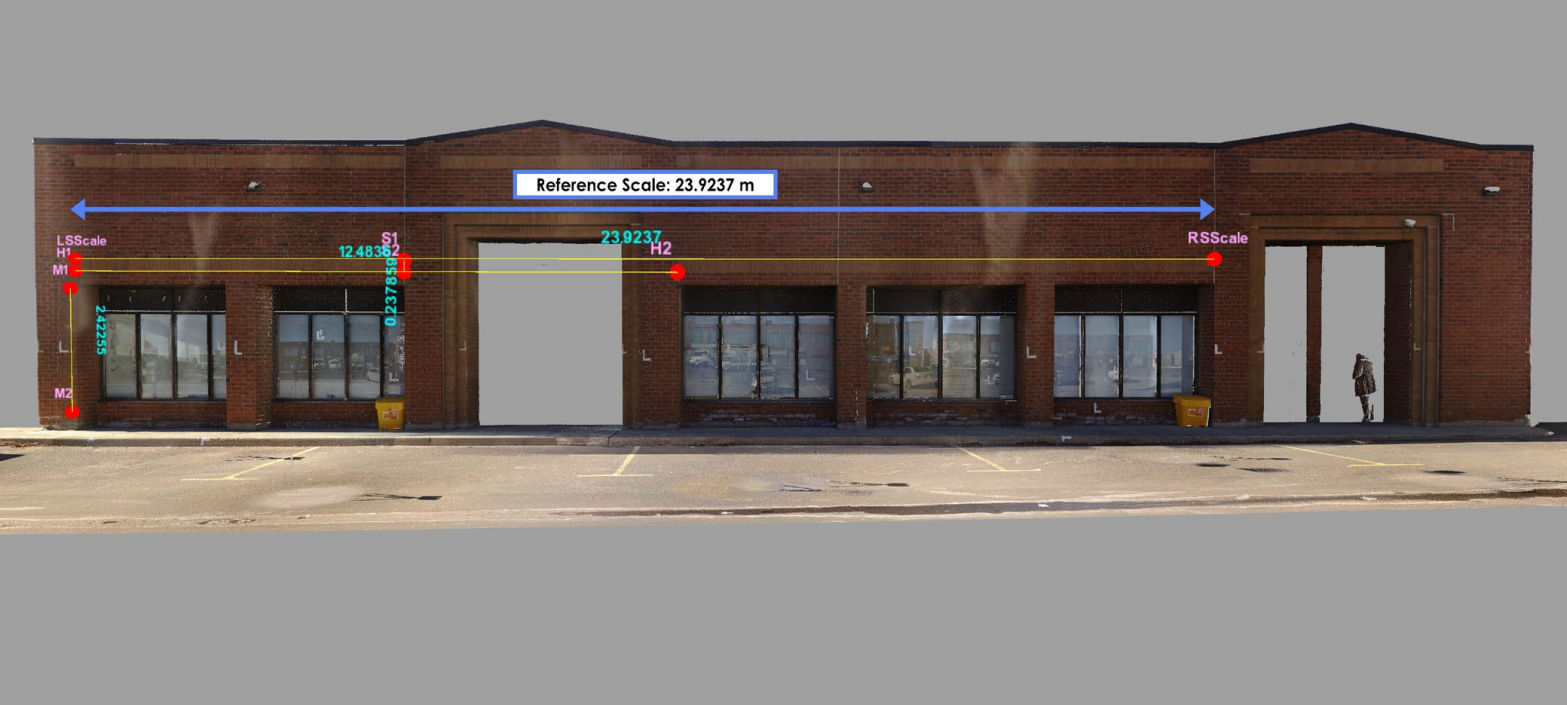
Reference scale measurement from the laser scanner ground truth model. The scale bar between points LSScale and RSScale spans 23.924 m, serving as the reference dimension for calibrating all reconstructed models. This longest distance was selected to reduce error propagation during scaling, as shorter reference distances would amplify measurement uncertainties. Control points mark the three validation distances: Long (H1‐H2: 12.48 m), medium (M1‐M2: 2.42 m), and short (S1‐S2: 0.24 m). All 30 reconstructed models from body‐worn camera video were scaled to match this 23.924 m reference, transforming unitless photogrammetric reconstructions into metrically accurate models. This scaling approach ensures consistent dimensional calibration across all camera models and resolutions, enabling direct comparison both among reconstructed models and with ground truth measurements.

3DF Zephyr cannot determine absolute scale from video reconstruction without reference measurements, necessitating manual scaling of all reconstructed models. Scaling required identifiable features that could be accurately and consistently located in both the reference model and all video reconstructions. The longest distance meeting these criteria spanned 23.924 m between two distinct architectural features designated as scale reference points (Figure 9). While not encompassing the entire building length, this distance was optimal for minimizing error propagation during scaling, as shorter reference distances would increase scaling uncertainties across the model. This reference distance encompassed all three validation measurements (12.48, 2.42, and 0.24 m) within its span, ensuring that any scaling errors would affect points beyond the reference span rather than the validation distances contained within it (Table 2). All 30 reconstructed models were scaled to match this 23.924 m reference dimension, converting dimensionless photogrammetric reconstructions into dimensioned models ready for direct comparison with ground truth measurements.

**TABLE 2:** Ground truth measurements established using Faro Focus S350 laser scanner. The reference scale (23.9237 m) represents the longest distance used to calibrate all reconstructed models. Three validation distances—long (12.4836 m), medium (2.4225 m), and short (0.2379 m)—were selected to assess measurement error across varying ranges. These point‐to‐point distances between control points serve as ground truth for quantifying measurement error across all camera models and resolutions. All values in m (precision: 0.1 mm) as measured; rounded to 2–3 decimal places elsewhere in manuscript.

Unit: Meter	Ground truth
Reference (scale)	23.9237
Long	12.4836
Medium	2.4225
Short	0.2379

**FIGURE 10:**
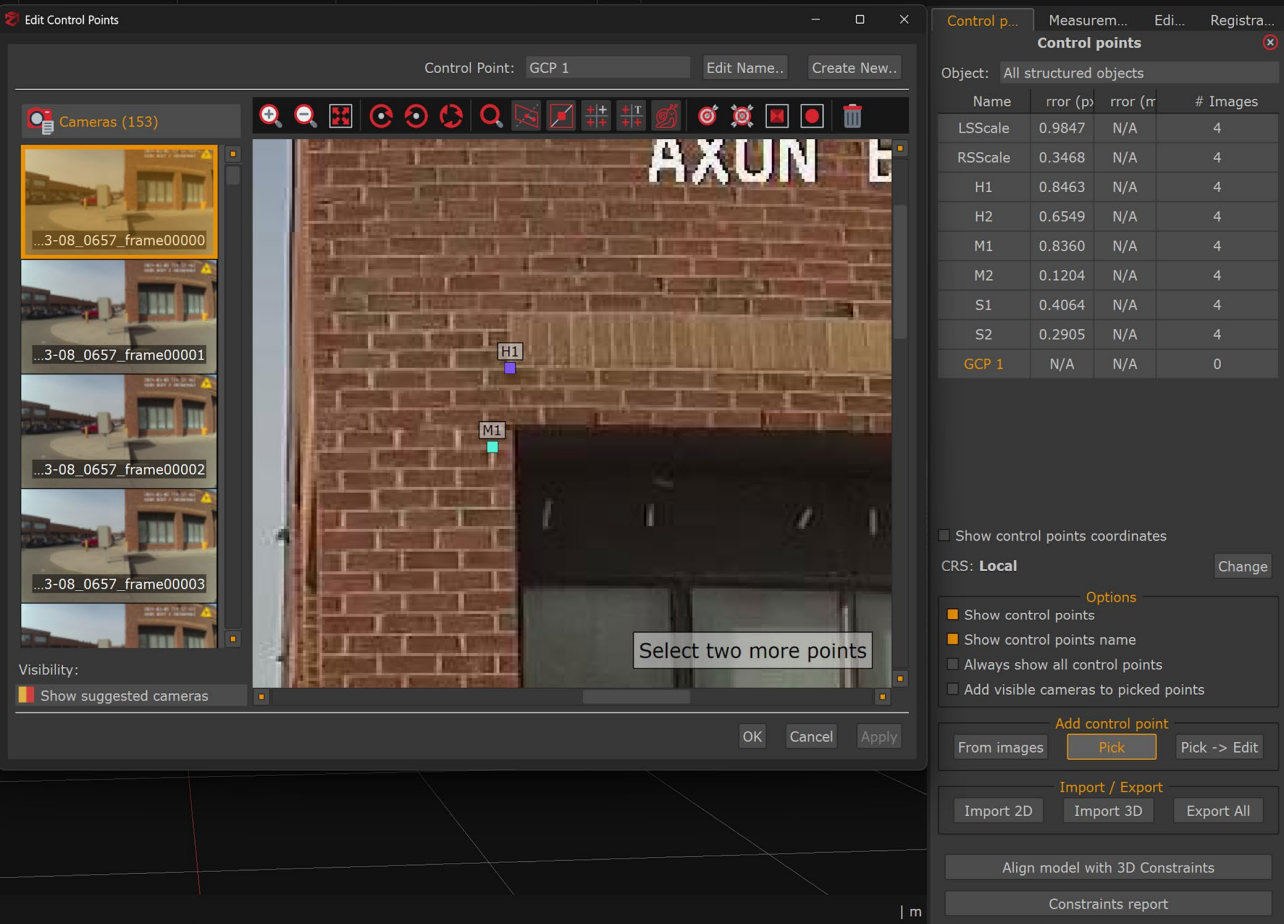
3DF Zephyr control point interface for manual selection of measurement references. The display shows M1 and M2 placement on distinct architectural features (brick pattern boundaries), demonstrating the selection process applied to all eight control points (LSScale, RSScale, H1, H2, M1, M2, S1, S2). Points were selected based on their visual distinctiveness and persistence across multiple frames to enable repeatable measurement. Control point pairs define the validation distances used to quantify measurement error both among the 30 reconstructed models and against laser scanner ground truth. Manual selection on identifiable features facilitates consistent distance measurement for accuracy validation against ground truth and performance comparison across camera models and resolutions.

**FIGURE 11:**
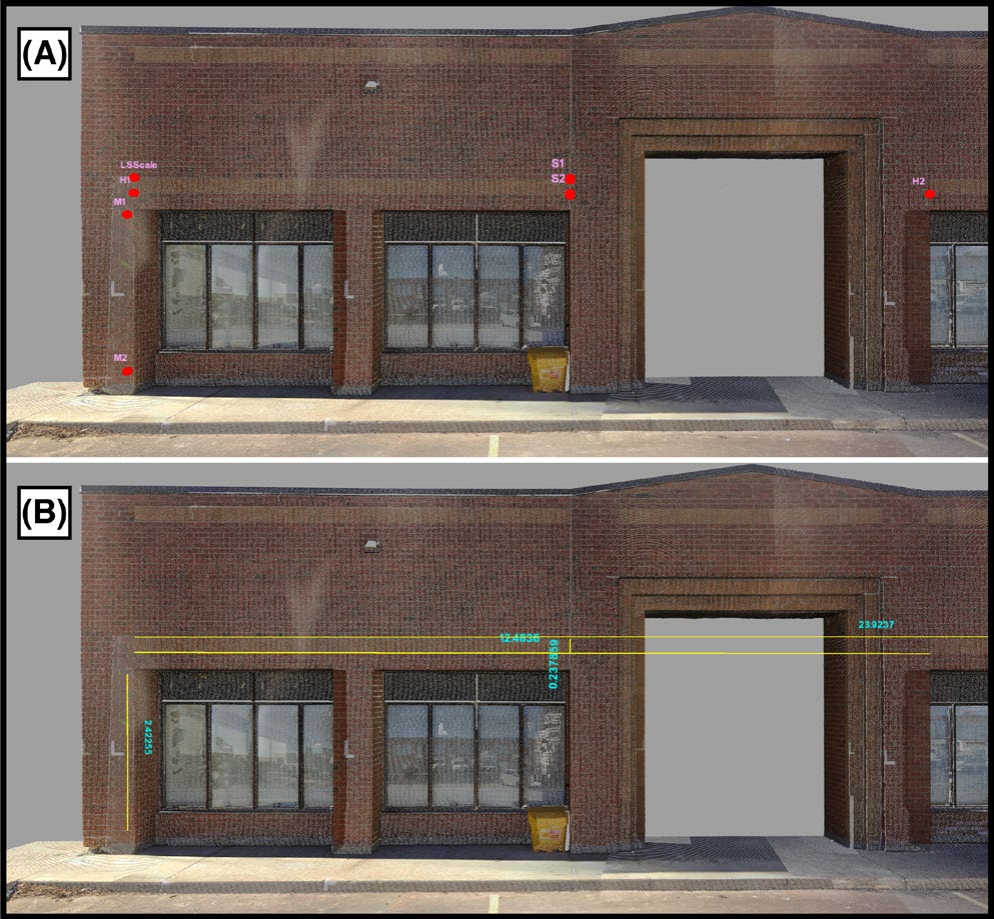
Laser scanner ground truth model showing control point placement and distance measurements. (A) Control points marked in red with pink labels on distinct architectural features of the building facade. (B) Distance measurements between control point pairs shown with yellow lines and blue labels displaying the measured values, where text orientation corresponds to measurement direction (horizontal text for horizontal distances, vertical text for vertical distances). The color coding and text alignment enhance clarity of control point locations and measurements that may be difficult to discern in previous figures.

**FIGURE 12:**
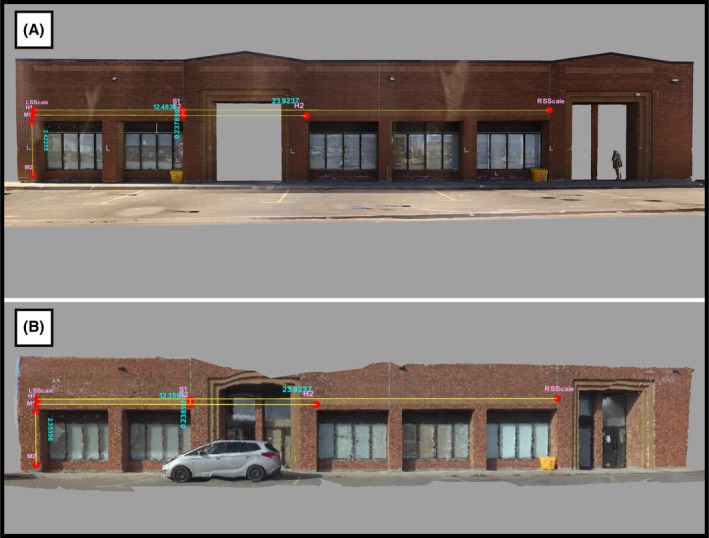
Comparison between laser scanner ground truth and photogrammetric reconstruction for measurement validation. (A) Laser scanner reference model with control points and distance measurements including the 23.924 m reference scale. (B) Dimensioned 3D reconstruction from AB2 camera at 720P resolution showing the same control points and measured distances. Identical control points on both models enable direct point‐to‐point distance comparison, where measurement differences quantify the error for this specific camera model and resolution.

After model scaling, measurement validation used point‐to‐point distance comparison to assess dimensional differences between reconstructed models and ground truth [18, 19]. Control points were placed at brick pattern intersections where horizontal and vertical mortar lines formed distinct cross‐patterns identifiable in the reference model and all video reconstructions. Each point was established in 3DF Zephyr by manually marking the feature across at least four overlapping frames that captured it from different viewing angles (Figure 10). The software then performed bundle adjustment, a non‐linear least‐squares optimization that calculates the 3D position minimizing reprojection error (the pixel distance between observed and projected point locations) across all marked frames [18, 19]. This process transforms multiple 2D pixel coordinates into a single 3D spatial coordinate, establishing the control point location. The measurement panel calculated distances between control point pairs at three ranges: long (H1–H2: 12.48 m), medium (M1–M2: 2.42 m), and short (S1–S2: 0.24 m) (Figure 11). These distances were selected to examine measurement accuracy across different scales. With identical point pairs measured in both the reference model and all reconstructions, this process generated 90 distance measurements (30 models × 3 distances) for comparison with ground truth (Figure 12). These point‐to‐point comparisons established the measurement error for each camera model‐resolution combination, providing the quantitative data for statistical analysis presented in the next section.

## 3 RESULTS

Three statistical tests applied to the 90 distance measurements revealed distinct patterns in dimensional reconstruction accuracy across validation distances, resolutions, and camera models.

One‐sample *t*‐tests (*α* = 0.05, two‐tailed) evaluated whether measurement errors (calculated as absolute difference between measured reconstruction distance and ground truth distance) differed significantly from zero (Tables 2 and 3). A non‐significant result (*p* ≥ 0.05) means the errors are close to zero, indicating accurate measurements. The AF2 camera demonstrated significant errors at long distances for both resolutions and at medium distances for 720P (Table 3). Long‐distance errors were 14.416 cm (*p* = 0.0004) at 720P and 7.312 cm (*p* = 0.0244) at 1080P for the 12.484 m reference distance. Medium distance errors reached 6.702 cm (*p* = 0.0016) at 720P and 2.246 cm at 1080P (*p* = 0.0688, non‐significance) for the 2.423 m reference distance. Short distance errors remained non‐significant for both resolutions: 0.452 cm (*p* = 0.3737) at 720P and 0.180 cm (*p* = 0.1150) at 1080P for the 0.238 m reference distance. This pattern indicates that AF2 dimensional reconstruction accuracy degraded with increasing measurement distance, with 720P resolution producing 2–3 times greater errors than 1080P across all distance categories. AB2 and AB3 cameras produced measurement errors not significantly different from zero across all conditions (all *p* ≥ 0.05). AB2 maximum error reached 5.498 cm at long distances with 1080P, while AB3 remained below 1.158 cm across all distance categories. Short distance errors for both cameras stayed below 0.508 cm regardless of resolution (Table 3). While all three camera models achieved centimeter‐level measurement precision, only AB2 and AB3 demonstrated errors statistically indistinguishable from ground truth across all validation distances, suggesting these models can produce dimensionally more accurate reconstructions from body‐worn camera video.

**TABLE 3:** Measurement errors across camera models and resolutions at three validation distances. Mean error (cm) calculated from five video reconstructions per camera‐resolution combination, using the absolute difference between measured and ground truth distances. One‐sample *t*‐tests (two‐tailed) evaluate whether mean absolute errors significantly differ from zero (*p* < 0.05 indicates significant measurement error; *p* ≥ 0.05 indicates error within random variation). AF2 camera showed significant measurement errors at long distances (1080P: 7.3120 cm, *p* = 0.0244; 720P: 14.4160 cm, *p* = 0.0004) and medium distance at 720P (6.7020 cm, *p* = 0.0016). AB2 and AB3 cameras showed no significant measurement errors (all *p* ≥ 0.05).

Unit: Centimeter	1080P	720P	1080P	720P	1080P	720P
*Long*	AB2	AB2	AB3	AB3	AF2	AF2
Mean error	5.4980	3.3340	0.6400	1.0480	7.3120	14.4160
*p*‐value	0.1041	0.4581	0.8011	0.3421	0.0244	0.0004
*Medium*	AB2	AB2	AB3	AB3	AF2	AF2
Mean Error	1.4820	0.1020	1.1580	0.6600	2.2460	6.7020
*p*‐value	0.6986	0.9584	0.2349	0.2277	0.0688	0.0016
*Short*	AB2	AB2	AB3	AB3	AF2	AF2
Mean error	0.3060	0.4540	0.0640	0.5080	0.1800	0.4520
*p*‐value	0.7413	0.0706	0.7495	0.1323	0.1150	0.3737

Two‐sample *t*‐tests (Welch's method, *α* = 0.05, two‐tailed) evaluated resolution effects on feature measurement accuracy by comparing 720P and 1080P measurements pooled across all camera models. No statistically significant differences were detected between resolutions at any validation distance: long (*p* = 0.8115), medium (*p* = 0.2798), and short (*p* = 0.1480) (Table 4). Mean differences between resolutions ranged from −0.71 cm to 1.78 cm. When compared against ground truth, 1080P produced smaller absolute errors than 720P at medium (0.64 cm vs. 2.42 cm) and short (0.02 cm vs. 0.47 cm) distances, while 720P performed better than 1080P at long distances (3.34 cm vs. 4.06 cm). Despite these numerical differences, the absence of statistical significance indicates that resolution had minimal practical impact on feature measurement accuracy within the controlled conditions and given internal software interpolation.

**TABLE 4:** Two‐sample *t*‐test (Welch's method) comparison of measured distances between video resolutions. Measurements pooled across all camera models (*n* = 15 per resolution: 5 videos × 3 cameras) to evaluate resolution effects. Mean differences calculated as 1080P minus 720P using signed values to preserve directional information. Welch's correction applied due to unequal variances between groups (variance ratios up to 2.6:1), producing adjusted degrees of freedom. No statistically significant differences detected at any validation distance (*p* = 0.8115, 0.2798, 0.1480 for long, medium, short distances; all two‐tailed tests). Mean differences ranged from −0.71 to 1.78 cm. Resolution did not significantly affect measurement accuracy within the controlled conditions and internal software interpolation.

	Long	Medium	Short
Mean 1080P (m)	12.4430	2.4161	0.2381
Mean 720P (m)	12.4502	2.3983	0.2332
Mean difference (m)	−0.0071	0.0178	0.0049
*T*‐Statistic	−0.2412	1.1028	1.4968
Degrees of freedom	23	27	23
*p*‐Value	0.8115	0.2798	0.1480

Single‐factor ANOVA revealed significant camera model effects on feature measurement accuracy at long (*F*(2,27) = 10.1851, *p* = 0.0005) and medium distances (*F*(2,27) = 4.5780, *p* = 0.0194), but not at short distances (*F*(2,27) = 0.1945, *p* = 0.8244) (Table 5). Effect sizes decreased with distance: 0.4300 (long), 0.2526 (medium), and 0.0142 (short), indicating that camera model differences had less pronounced effects at shorter measurement ranges. Post‐hoc Tukey HSD tests (*α* = 0.05) identified AF2 as significantly different from both AB2 (*p* = 0.0043) and AB3 (*p* = 0.0010) at long distances, and from AB2 (*p* = 0.0167) at medium distances. No significant differences were detected between AB2 and AB3 at any distance (*p* ≥ 0.05), confirming comparable performance between these two camera models. These results indicate that camera model selection affects dimensional reconstruction accuracy at longer measurement distances, with AF2 showing suboptimal performance compared to AB2 and AB3 despite standardized experimental conditions and identical software processing.

**TABLE 5:** Single‐factor ANOVA examining camera model effects on measured distances at three validation ranges. Analysis performed with measurements pooled across resolutions (*n* = 10 per camera: 5 videos × 2 resolutions). Significant camera effects detected at long (*F*(2,27) = 10.1851, *p* = 0.0005) and medium distances (*F*(2,27) = 4.5780, *p* = 0.0194), but not at short distance (*F*(2,27) = 0.1945, *p* = 0.8244). Post‐hoc Tukey HSD tests identified AF2 as significantly different from both AB2 and AB3 at long distance, and from AB2 at medium distance. Effect sizes (0.4300, 0.2526, 0.0142 for long, medium, short respectively) indicate the proportion of variance attributable to camera model decreases with distance.

Distance	Camera	Mean ± SD (m)	*F*(2,27)	*p*‐value	Effect size	Pairwise comparisons
Long	AB2	12.4728 ± 0.0858	10.1851	0.0005*	0.4300	AB2 vs. AB3: ns (*p* = 0.7550)
	AB3	12.4920 ± 0.0384				AB2 vs. AF2: * (*p* = 0.0043)
	AF2	12.3750 ± 0.0526				AB3 vs. AF2: * (*p* = 0.0010)
Medium	AB2	2.4304 ± 0.0602	4.5780	0.0194*	0.2526	AB2 vs. AB3: ns (*p* = 0.6036)
	AB3	2.4134 ± 0.0144				AB2 vs. AF2: * (*p* = 0.0167)
	AF2	2.3778 ± 0.0301				AB3 vs. AF2: ns (*p* = 0.1297)
Short	AB2	0.2372 ± 0.0138	0.1945	0.8244	0.0142	AB2 vs. AB3: ns (*p* = 0.8641)
	AB3	0.2350 ± 0.0054				AB2 vs. AF2: ns (*p* = 0.8236)
	AF2	0.2347 ± 0.0070				AB3 vs. AF2: ns (*p* = 0.9000)

*Note*: Values presented as mean ± standard deviation. Effect size calculated as proportion of variance explained (SSbetween/SStotal). Pairwise comparisons performed using post‐hoc Tukey HSD test (*α* = 0.05). Shaded cells marked with asterisks (*) indicate statistical significance (*p* < 0.05) for overall ANOVA and pairwise comparisons; ns = not significant (*p* ≥ 0.05).

Overall, the three statistical analyses revealed statistically significant variation in dimensional reconstruction accuracy by camera model but not by recording resolution under controlled experimental conditions with internal software interpolation. All three camera models achieved centimeter‐level measurement precision, yet only AB2 and AB3 produced measurements statistically indistinguishable from ground truth across all validation distances. The AF2 camera showed significant measurement errors that increased with distance, reaching 14.416 cm mean error at long distance 720P recordings. Recording resolution (720P vs. 1080P) produced no statistically significant effects on feature measurement accuracy across any camera model or validation distance. This absence of resolution effects, despite theoretical expectations of improved accuracy with higher pixel density, suggests that the software‐specific reconstruction algorithms and internal processing methods could have compensated for resolution differences in an effective manner. Camera models emerged as the primary factor affecting dimensional reconstruction accuracy. The narrower field of view of AF2 (120°) corresponded with greater measurement errors compared to the wider fields of view of AB2 (143°) and AB3 (146°), indicating that optical characteristics and field of view specifications influenced model performance more than pixel density in this study. These findings establish baseline feature measurement accuracy achievable from body‐worn camera video when processed through automated photogrammetric calibration under optimized recording conditions and identical software processing procedures. The quantitative accuracy profiles documented here provide empirical data for assessing the feasibility and limitations of dimensional reconstruction from body‐worn camera video for forensic applications.

## 4 DISCUSSION

Beyond the quantitative measurements and statistical results, this section examines the broader implications of these findings for forensic applications and identifies methodological constraints that affected dimensional reconstruction accuracy from body‐worn camera video using 3DF Zephyr. The results reveal both expected limitations and unexpected compensatory mechanisms that influence the feasibility of transforming body‐worn camera video into measurable 3D reconstructions. While statistical analysis demonstrated significant camera model effects but no significant resolution effects, understanding these patterns requires examining the interaction between hardware specifications, video compression artifacts, and photogrammetric processing algorithms. The following discussion addresses four key aspects: (1) how controlled data collection conditions established baseline reconstruction capabilities while deviating from field realities, (2) how video compression challenges were mitigated through camera movement providing new spatial information in each frame, (3) how photogrammetry software processing and automated calibration affected accuracy compared to potential alternative approaches, and (4) how these findings inform practical forensic implementation. These considerations help establish whether body‐worn cameras can transition from qualitative documentation tools to quantitative measurement instruments suitable for forensic reconstruction and legal proceedings.

### 4.1 Controlled data collection and field reality deviation

The recording methodology optimized conditions in a deliberate manner to establish baseline dimensional reconstruction feasibility, creating a significant departure from field documentation scenarios. The outdoor parking lot selection with favorable lighting, steady sideways movement along a predetermined 30 m path, and multiple recording attempts to obtain optimal video quality represent best‐case scenarios absent in actual crime scene response. Officers encounter time constraints, unpredictable environments, safety priorities, and single‐opportunity documentation that preclude controlled procedures. However, this best‐case approach remains methodologically necessary to determine whether body‐worn cameras possess fundamental technical abilities for accurate dimensional reconstruction. If accurate reconstructions cannot be achieved under optimal conditions, field applications would be infeasible. The successful reconstructions across all three camera models confirm that the technology possesses fundamental technical abilities for accurate dimensional reconstruction under controlled conditions, though accuracy varied from centimeter‐level precision with AB2 and AB3 to decimeter‐level with AF2.

Camera hardware specifications introduce variations that corresponded with observed dimensional reconstruction accuracy differences. Field of view variation between camera models affects frame overlap and tie point availability, explaining the observed performance disparities. The wider fields of AB2 (143°) and AB3 (146°) capture more comprehensive scene coverage, providing greater frame overlap essential for tie point matching across frames. While these wider views introduce greater barrel distortion requiring geometric correction during processing, the increased overlap and tie point density compensate for distortion‐related uncertainties. The narrower 120° field of AF2 reduces optical distortion but limits scene coverage per frame, compromising performance at long distances where sufficient frame overlap becomes critical for accurate triangulation. The maximum mean error of 14.42 cm at 720P for AF2 corresponds to this reduced field of view limiting tie point availability at greater measurement distances. Hardware design differences further compound field of view effects. The AF2 modular design allows camera head movement independent of the controller unit, introducing positioning variability during recording due to challenges in maintaining stable manual control. This variability, absent in the chest‐mounted integrated units of AB2 and AB3, creates inconsistent camera trajectories that compromise bundle adjustment calculations. Firmware version inconsistencies within AF2 units (0.5.9 and 0.7.3) introduce additional performance variation not present in the consistent firmware implementations of AB2 (1.27.7) and AB3 (1.19.45). These combined hardware and firmware variations could have contributed to the significant measurement errors observed in AF2, while AB2 and AB3 produced measurements statistically indistinguishable from ground truth across all resolutions and validation distances.

Future field implementation would benefit from bridging the gap between controlled conditions and operational realities. Officers could receive training in photogrammetric documentation principles, including walking speeds that maintain substantial frame overlap (60–80%), consistent camera‐to‐subject distances to prevent scale variations, and systematic movement patterns for comprehensive scene coverage [20, 21, 22]. Based on photogrammetric principles, documentation protocols could recommend limiting viewing angle variations to 30° between consecutive positions to preserve tie point quality [20, 21, 22]. Photogrammetric training exercises might incorporate various challenging conditions encountered at crime scenes: low‐light environments where feature visibility is reduced, reflective surfaces where spurious points create geometric distortions, and uniform surfaces where texture‐based matching fails. Adopting standardized firmware versions across camera units and maintaining consistent chest‐mounted positionings could reduce the hardware‐related variations identified in this study.

### 4.2 Video compression challenges and movement compensation

Video compression creates frame type variability that requires examination for photogrammetric reconstruction applications. Axon body‐worn cameras encode video with a GOP structure of 30 frames: one I‐frame containing complete image data followed by 29 P‐frames storing differential information relative to preceding frames [15]. When 3DF Zephyr extracts frames at 3 frames per second from these videos, the software does not differentiate between frame types, producing datasets with mixed I‐frame and P‐frame distributions in unknown proportions. The video decoder reconstructs P‐frames into complete images before extraction by referencing their temporal dependencies within the video file, ensuring photogrammetry software receives fully decoded frames rather than differential data. However, P‐frames accumulate compression artifacts through prediction error propagation across temporal encoding chains, while I‐frames maintain higher spatial fidelity through independent encoding [15]. This quality differential could affect feature detection and tie point matching precision during photogrammetric reconstruction.

Nevertheless, the controlled camera movement pattern in this study compensated for potential compression‐related quality degradation. Continuous lateral translation along the building facade ensured each extracted frame captured different viewing angles regardless of whether 3DF Zephyr selected I‐frames or P‐frames at extraction intervals. This movement pattern meant P‐frames documented substantial new spatial information through perspective changes rather than minimal variations within static viewpoints. The consistent walking speed generated regular baseline distances between consecutive frames, providing adequate overlap for tie point identification and matching despite any compression artifacts present in P‐frame data. This spatial compensation mechanism explains reconstruction success across all three camera models despite mixed frame type distributions and absent frame type control during extraction.

The decision to process videos without pre‐extraction compression analysis reflected practical constraints appropriate for this validation. Pre‐extraction frame type identification requires video analysis tools such as ffprobe to parse encoding metadata and catalog I‐frame positions within GOP structures. Selective I‐frame extraction would provide frames encoded with complete spatial information at maximum available quality. However, the measurement accuracy achieved across camera models and resolutions at all validation distances suggests compression artifacts from P‐frame inclusion did not prevent centimeter‐level dimensional reconstruction. The differential performance observed between camera models under identical compression handling and frame extraction parameters indicates hardware specifications influenced reconstruction outcomes more than frame type distribution when continuous perspective changes were present.

Future forensic applications could investigate whether GOP structure optimization produces measurable accuracy improvements beyond current performance levels. Frame type cataloging before extraction would enable selective I‐frame processing or extraction rate alignment with GOP boundaries to control frame type ratios. Such optimization may reduce compression artifact effects on feature detection quality. However, the primary consideration for field implementation should focus on documentation movement patterns rather than compression analysis protocols. The compensatory effect of continuous lateral camera motion proved sufficient to achieve acceptable dimensional reconstruction accuracy despite uncontrolled frame type distributions, suggesting deliberate movement protocols may provide more practical accuracy improvements than preprocessing complexity for forensic reconstruction applications.

### 4.3 Photogrammetric processing and software dependencies

The photogrammetric reconstruction process incorporates manual operations and software‐specific procedures that introduce variability affecting the measurement accuracy documented in this validation. Manual control point selection required the researcher to identify brick pattern intersections across at least four overlapping frames per point, marking pixel locations that 3DF Zephyr then used for bundle adjustment calculations. This manual marking process introduces positioning uncertainty where the researcher interprets which pixel coordinates represent the true feature location within each frame. Architectural features with gradual edges rather than sharp boundaries increase interpretation variability. Different researchers with varying experience levels would mark different pixel locations for the same physical feature. The study used a single researcher for internal consistency but did not assess inter‐researcher reliability that would affect forensic applications involving multiple technicians. Software parameter selection represents another manual decision point affecting reconstruction outcomes. The study applied identical 3DF Zephyr settings across all 30 videos, with parameters optimized for large architectural features. These parameter choices represent only one possible configuration. Alternative parameter combinations might produce different accuracy profiles. For instance, higher extraction rates would capture more frames but increase processing time and potentially introduce redundant data. Lower noise filtering percentages would retain more surface detail but include more outlier points requiring manual removal. Different watertightness values would alter surface continuity in reconstructed models. The automated calibration process itself implements optimization algorithms that minimize reprojection errors across extracted frames, and these algorithms function as black‐box operations where the researcher cannot verify intermediate calculations or adjust convergence criteria. These manual operations and parameter choices compound with hardware specifications and processing procedures to produce the measurement errors documented in this validation.

This validation scope remains restricted to Axon body‐worn camera video processed through 3DF Zephyr using the documented procedures, where measurement errors reflect this specific combination of hardware, software, and researcher decisions. The measurement accuracy established in this study provides a reference baseline and achievable range for forensic applications rather than absolute minimum or maximum performance limits. The single‐researcher design provides no data on whether forensic technicians with different experience levels would achieve comparable accuracy from identical video datasets. The controlled conditions and deliberate parameter selection enhanced reconstruction quality, yet alternative parameter combinations may achieve lower or higher measurement errors. These documented accuracy levels represent 3DF Zephyr‐specific performance rather than generalizable photogrammetric capabilities. The non‐significant resolution effects between 720P and 1080P may reflect software‐specific interpolation, and the camera model dependencies where AF2 demonstrated greater errors than AB2 and AB3 could differ under alternative photogrammetry platforms implementing different lens distortion correction algorithms.

Addressing these software‐specific and researcher‐dependent constraints requires systematic investigation across multiple variables beyond this baseline validation. Four future research directions would address current limitations: (1) testing reconstruction accuracy across multiple photogrammetry software platforms would distinguish software‐specific performance from methodology‐specific constraints, (2) conducting inter‐researcher reliability studies with forensic technicians at different experience levels processing identical video datasets would quantify measurement variability, (3) performing parameter sensitivity analysis would establish how extraction rates, noise filtering thresholds, and calibration methods affect measurement accuracy, and (4) executing field validation using actual crime scene video under operational constraints rather than controlled test conditions would assess operational feasibility. These systematic investigations would establish evidence‐based processing standards and training protocols for first responders and forensic technicians. Training programs could then incorporate validated best practices for camera movement patterns, software parameter selection, and control point identification procedures specific to body‐worn camera reconstruction. Standardized protocols emerging from such investigations would transform body‐worn cameras from passive documentation devices into validated measurement instruments accepted in legal proceedings, enabling dimensional evidence presentation beyond current qualitative video review.

### 4.4 Implementation limitations and practical considerations

The measurement errors documented in this validation must be evaluated against application‐specific accuracy requirements that vary across forensic contexts. Different measurement scales demand different error tolerances. For room‐scale architectural measurements spanning 10–15 m, centimeter‐level errors represent fractional percentage deviations that preserve spatial relationships. For suspect height estimation spanning 1.5–2 m, the same centimeter‐level errors constitute proportionally greater uncertainty affecting identification reliability. For bloodstain pattern analysis examining features at millimeter scale, centimeter‐level errors exceed dimensional tolerances necessary for trajectory reconstruction. This validation cannot determine which error magnitudes remain acceptable for specific forensic applications, as such determinations depend on case‐specific requirements, legal standards, and expert interpretation that vary across jurisdictions and contexts. The documented accuracy profiles establish what measurement precision body‐worn camera reconstruction achieves under tested conditions, not what precision different forensic applications require.

This study validated dimensional reconstruction accuracy for large architectural features based on practical considerations of body‐worn camera positioning and movement during scene documentation. Cameras mounted at chest height capture scenes at distances determined by officer position relative to architectural elements, producing video documentation of room‐scale spatial relationships rather than close‐range evidence details. This validation approach reflects operational reality where body‐worn cameras document overall scene context during initial response, while close‐range evidence photography follows separate protocols. The focus on large‐scale features does not establish that smaller evidence items cannot be reconstructed from body‐worn camera video, but rather that such reconstruction was not validated here and would require separate testing under different recording conditions. Close‐range reconstruction would demand different camera‐to‐subject distances, potentially different resolution settings, and alternative control point selection strategies than those validated for architectural feature measurements. Whether body‐worn cameras achieve comparable accuracy for small evidence reconstruction remains an empirical question requiring dedicated validation in future research rather than extrapolation from architectural feature performance.

In conclusion, this controlled validation provides the initial systematic empirical assessment of dimensional reconstruction accuracy from body‐worn camera video for forensic applications. AB2 and AB3 cameras achieved measurement errors statistically indistinguishable from ground truth across all validation distances, demonstrating that body‐worn cameras can preserve dimensional information during immediate crime scene capture, enable large‐scale feature measurement for spatial relationship documentation, and provide quantifiable data for temporal scene comparison. AF2 performance limitations establish that camera model selection affects reconstruction accuracy under identical processing conditions. These baseline accuracy profiles enable forensic practitioners to evaluate whether specific applications fall within validated accuracy boundaries or require alternative documentation methods. The controlled conditions and 3DF Zephyr‐specific processing establish performance under favorable circumstances rather than operational constraints. Field implementation requires systematic investigation of the hardware dependencies, software constraints, and operational factors identified here. This baseline validation establishes fundamental technical abilities for accurate body‐worn camera dimensional reconstruction while providing empirical foundations for developing camera selection guidelines, processing standards, and application‐specific protocols that would transform body‐worn cameras from qualitative documentation devices into quantitative measurement tools for forensic applications.

## CONFLICT OF INTEREST STATEMENT

The authors declare that there are no financial or personal interests that could have been perceived as influencing the research presented.

## Data Availability

The data that support the findings of this study are available from the corresponding author upon reasonable request.
